# Undoing the ‘Nordic Paradox’: Factors affecting rates of disclosed violence against women across the EU

**DOI:** 10.1371/journal.pone.0249693

**Published:** 2021-05-05

**Authors:** Anne Laure Humbert, Sofia Strid, Jeff Hearn, Dag Balkmar

**Affiliations:** 1 Centre for Diversity Policy Research and Practice, Oxford Brookes Business School, Oxford Brookes University, Oxford, United Kingdom; 2 Division of Gender Studies and Sociology, School of Humanities, Education and Social Sciences, Örebro University, Örebro, Sweden; 3 Department of Management and Organisation, Hanken School of Economics, Helsinki, Finland; 4 Department of Human and Health Sciences, University of Huddersfield, Huddersfield, United Kingdom; 5 Institute for Social and Health Sciences, University of South Africa, Lenasia, Republic of South Africa; South African Medical Research Council, SOUTH AFRICA

## Abstract

Measuring violence against women raises methodological questions, as well as the wider question of how to understand violence and locate it in relation to a societal context. This is all the more relevant given that measurement of violence against women in the EU has made an interesting phenomenon apparent, the so-called ‘Nordic Paradox’, whereby prevalence is higher in more gender equal countries. This article examines this phenomenon by exploring a range of factors—methodological, demographic and societal—to contextualise disclosed levels of violence. The analysis makes use of a multilevel analytic approach to take into account how macro and micro levels contribute to the prevalence of violence. The intercepts are then used to illustrate how taking these into account might provide an alternative ranking of levels of violence against women in EU countries. The results show that the ‘Nordic Paradox’ disappears—and can be undone—when factors at individual and country levels are considered. We conclude that the ‘Nordic Paradox’ cannot be understood independently from a wider pattern of violence in society, and should be seen as connected and co-constituted in specific formations, domains or regimes of violence. Our results show that the use of multi-level models can provide new insights into the factors that may be related to disclosed prevalence of violence against women. This can generate a better understanding of how violence against women functions as a system, and in turn inform better policy responses.

## Introduction

As elsewhere across the world, violence against women is a critical issue across the EU, with on average at least one in three women experiencing violence over their lifetime [1]. However, assessing the scale, spread and impact of the problem more precisely is far from easy and straightforward. This is partly a methodological question of measurement of what is to count as violence and as violence against women, and whether this measurement is to be done by criminal statistics, social surveys, in-depth studies, and so on [2]. It is also a much wider question of how to understand violence against women, and violence more generally, and how to locate that understanding in relation to broad questions of societal context, and indeed politics and policy [3].

On the first methodological question, data come from a variety of sources. National and international initiatives provide statistical measurements of violence, with two main types of data sources. The first are administrative data sources, for example, from the police, judiciary or hospital records. However, lack of harmonisation, uneven protocols and low data collection hinder the quality of the data and their comparability across countries. The second data source is prevalence surveys, such as the EU-wide survey on violence against women carried out by the European Union Agency for Fundamental Rights (FRA) in 2012 with over 42,000 women respondents [1, 4]. This survey aimed to harmonise definitions and methodologies, but debates have surfaced as to whether it captures actual prevalence rates or only disclosed rates of violence from the respondents [5]. Furthermore, and what is especially important for this paper, is that data from the EU-wide survey interviews report higher prevalence of gender-based violence against women in countries that are typically associated with greater gender equality, the so-called ‘Nordic Paradox’ [6–8]. This calls for more research into this paradox to advance our knowledge on what determines individual risk of violence within and between countries, for policy-making, and to improve prevention initiatives.

Growing debates about the ‘Nordic Paradox’ examine the interpretation of these data, including: the extent to which questions about violence, definitions of violence and violent experiences have the same meanings in different national and linguistic contexts [9]; the extent to which violence, or rather different kinds of violence, are accepted and normalised [6, 10]; and the extent to which responses of exposure to violence are affected by social shame [11, 12]. It also raises questions about how to understand violence against women, and violence more generally in relation to societal context, and poses the very question of “what is violence?” in an even more fundamental way [13, 14]. The societal contextualising of violence and violence against women problematises any simple definition of violence and its boundaries [2, 15–17]. Violence is still often framed and defined in terms of physical violence, even to the extent that sometimes (physical) sexual violence is separated from physical violence and not even discussed as part of physical violence. Feminist activists and scholars have long argued that domestic violence, gender-based violence and intimate partner violence also include and entail what are (initially at least) non-physical types of violence (such as economic, psychological and emotional types of violence) [18, 19]. This sits along other types of violence and abuse around control of family and friendship, social life beyond the immediate relationship, animals and pets and technologies (such as mobile phones), and the societal, institutional and organisational arrangements that affect and construct such situations, events and eventualities [20–22]. Accordingly, violence and violence against women need to be understood in relation to societal conditions, broadly based structures of inequality, governance and welfare state regimes, as well as social movements. For example, great inequalities and entrenched oppressions can mean that the enactment of violence, especially physical violence, may not be immediately necessary to maintain oppressive or unequal social relations, when that threat of violence is available [21], such as in cases of symbolic violence [23] and structural violence [24]. Paradoxically, violence, or at least direct, interpersonal and physical violence, may not be used as necessary in some very violating contexts.

In this paper, we seek to examine the ‘Nordic Paradox’ by exploring how a range of factors—methodological, demographic and societal—can contextualise disclosed levels of violence. Responding to calls to use multi-level models [6, 7], we examine data from the FRA’s EU-wide survey on violence against women in relation to levels of gender equality as measured by EIGE’s Gender Equality Index using an analytic approach to take into account how macro and micro levels contribute to the production of deadly and damaging types of violence [3]. We then use the intercepts of this multi-level model as ‘corrected’ indicators of the average level of violence against women in respective countries. These, in turn, can be used to provide an alternative ranking of levels of violence against women in EU countries, and show how the ‘Nordic Paradox’ can be undone. We discuss the extent to which this alternative ranking relates (or not) to the Violence Regimes typology, and specifically the Violence Regimes Index [25]. Finally, we draw out methodological, theoretical and policy implications.

## Explaining differences in the prevalence of violence against women in the EU

**Fig 1:**
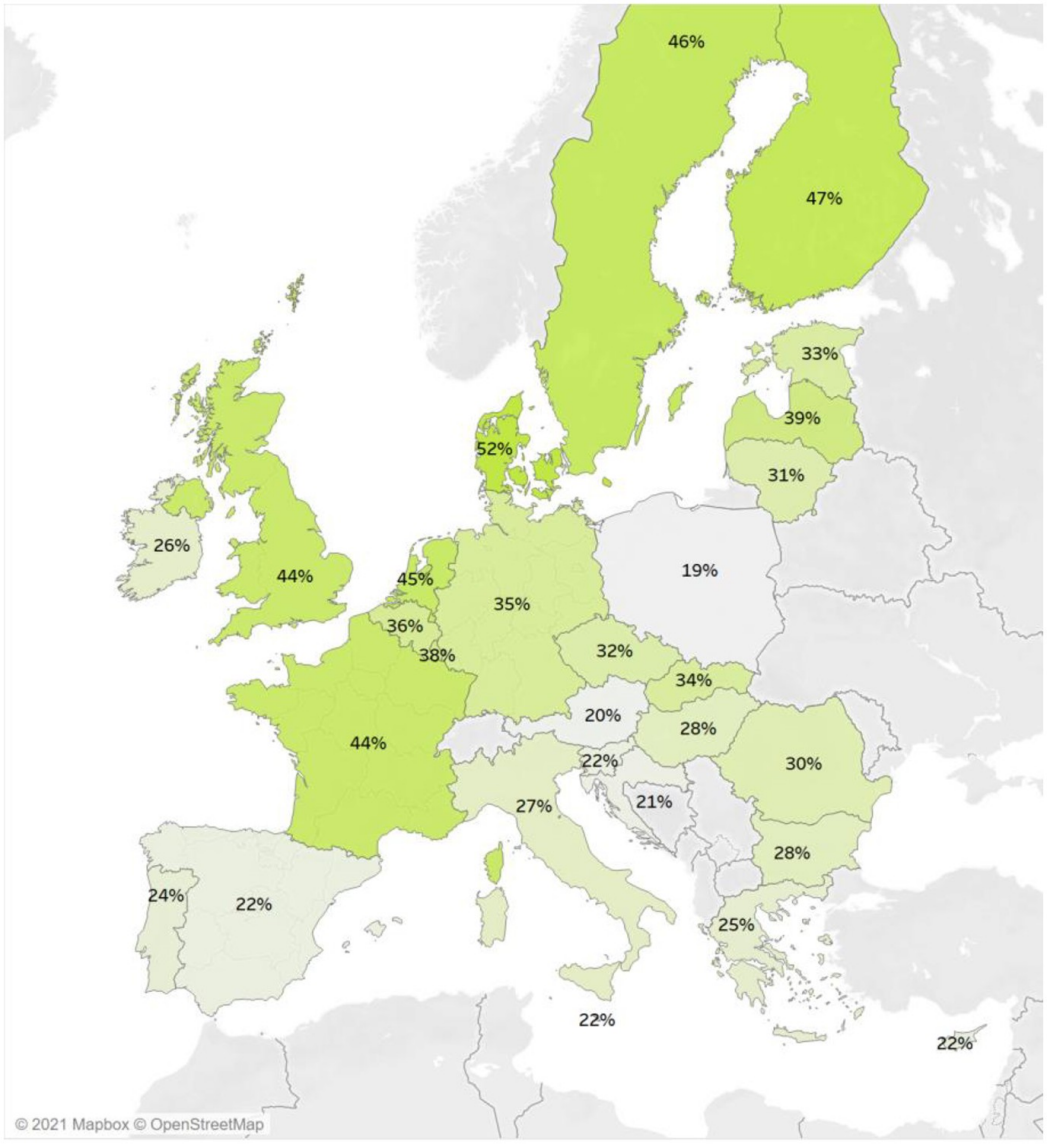
Disclosed prevalence of violence by Member State in 2012 (n = 41,954). Source: based on data from EU-wide Survey on Violence Against Women Note: EU average 32%.

According to FRA’s EU-wide Survey on Violence against Women [1] conducted in 2012, nearly one in three (33%) women in the EU have been a victim of violence against women in their lifetime since the age of 15 (Fig 1). This estimate is in line with previous surveys which often put lifetime prevalence of violence against women, by intimate partners or in a wider sense, around 30% [26–29] although this can range widely from 10 to 69% [30].

Conceptually, the case has been made that violence against women transcends cultural, and thus national, contexts [31]. Yet, empirically, total lifetime prevalence in the FRA survey ranges from 19% of women in Poland to 46% in Sweden, 47% in Finland and 52% in Denmark. The results thus show that there are higher prevalence rates in the Nordic countries, traditionally associated with greater gender equality than other countries [11]. High prevalence in Nordic Countries is consistent across different surveys measuring violence against women, such as the results of the Swedish National Council for Crime Prevention [32] or that of Wijma and colleagues [33]. Yet, these findings are at odds with earlier studies conducted at the global level, and which suggested that violence against women decreases as societal norms become more gender egalitarian [34]. They are also at odds with findings at the state level in the US, which have shown that higher gender inequalities are associated with higher prevalence of different types of violence against women [35, 36]. As Kearns and colleagues [37] argue, there is a complex relationship between violence against women and gender equality, and the possibility of a backlash effect needs to be considered. This means that even though greater gender equality might decrease the prevalence of violence against women, this is only true up to the point where social norms are challenged and men lose their traditionally dominant position in society [6]. For example, Kearns and colleagues [37] found that US states where there was a greater number of women represented in government also had higher prevalence of certain forms of sexual violence. The ‘Nordic Paradox’ phenomenon thus demonstrates the necessity to understand how gender relations are constructed in relation to spatiality [38].

The ‘Nordic Paradox’ can be better understood by looking at how differences in the prevalence of violence against women might arise from different sources, including methodological, demographic and societal factors. The Fundamental Rights Agency itself provides five possible explanations, highlighting the need for further exploration: acceptability of talking to other people about experiences of violence; increased awareness about violence; exposure to risk factors outside the home, such as being in employment; levels of violent crime; and alcohol consumption [1]. These explanations are far from exhaustive, and are largely based on earlier models of the ecological landscape of violence against women [e.g. 30]. One of the earliest and most influential frameworks is that of Heise [39], which presents violence against women as a multi-faceted phenomenon that has its origins in the interplay of personal, situational and socio-cultural factors. Yet, thus far, a limitation of research into understanding variations at national level has been that the focus has been on the number and scope of the factors considered, which disproportionately looked at the individual level, rather than the community or societal levels [30].

**Fig 2:**
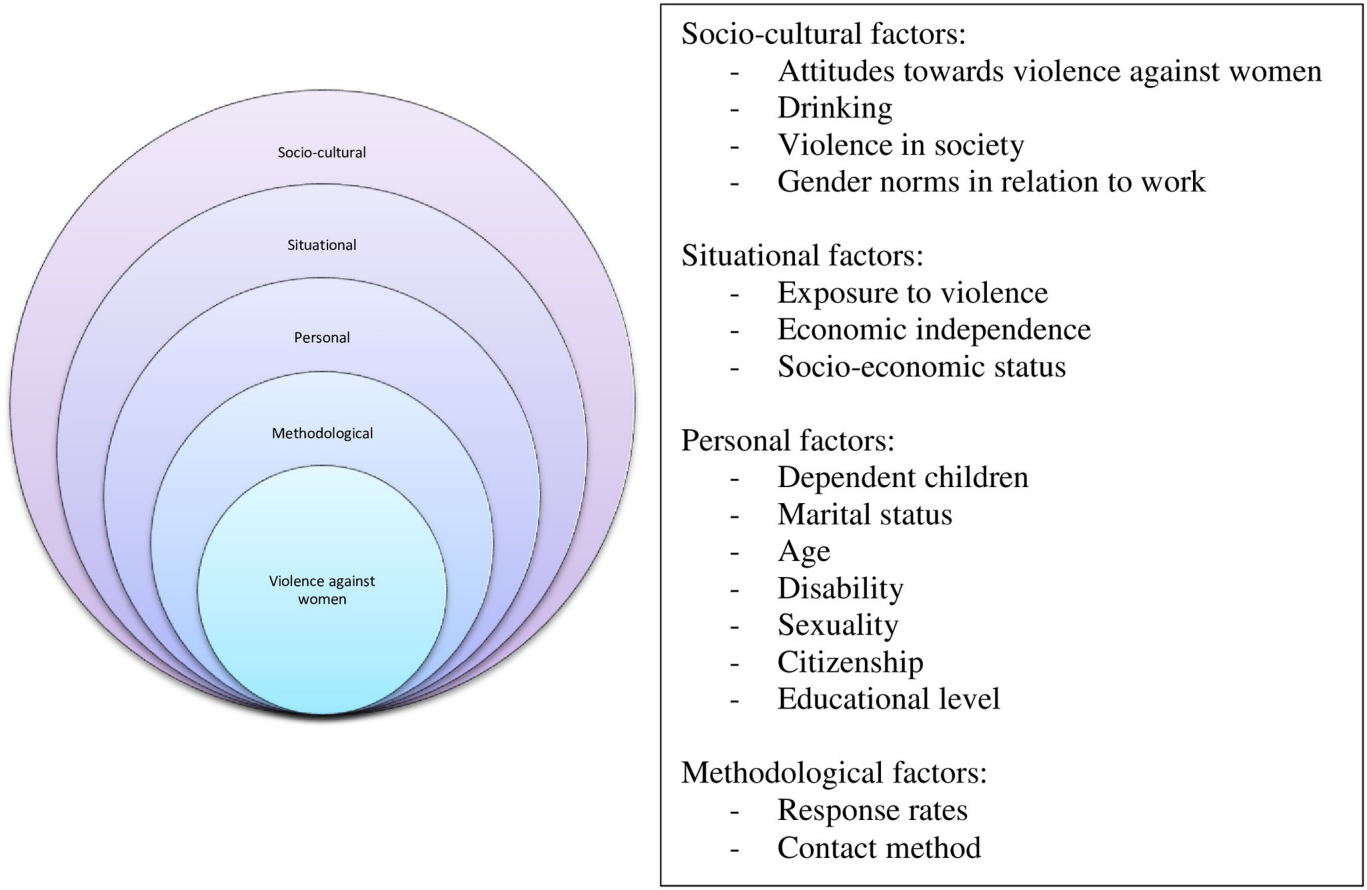
Multi-level factors affecting violence against women. Source: based on Heise (1998).

We build on Heise’s model and discuss in greater detail the potential effects of some factors on the disclosed prevalence of violence against women (Fig 2), in our efforts to better understand the ‘Nordic Paradox’. The choice of factors is not an exhaustive one, and we recognise that other factors may be of importance. We also do not make any claims as to either the directionality or the causality of any relationship between these factors and violence against women. We orient our selection using two main sources: FRA’s initial proposal to contextualise levels of violence throughout the EU, complemented by factors that have been mentioned in the literature on violence against women. We rely on data measuring outcomes (e.g. crime rates) and combine it with data on attitudes (e.g. towards violence against women). Our overall aim is to understand, whether once these factors have been taken into account, the ‘Nordic Paradox’ persists or whether it becomes undone.

### Socio-cultural factors

We consider the factors of potential relevance to explain levels of disclosed prevalence of violence among women. At the outset, it is important to understand that violence can be seen as a way to uphold gender norms in society [8, 19, 40]. Research, for example, has shown a positive correlation with gender inequalities, e.g. as measured by composite indicators such as the UNDP’s Gender Inequalities Index, and different measures of violence against women [34]. These measures rely on the aggregation of several domains of relevance to gender equality, such as for example in the case of the Gender Equality Index, inequalities in economic participation, political/economic representation or earnings [41]. Gender norms concern both notions of masculinities and femininities. Masculinities and violence interact in a complex way. While violence can be a way to assert a certain form of masculinity, being a ‘real man’ is also not resorting to violence [42, 43]. Femininities play a role in that women are expected to be ‘good’, ‘credible’ victims, and violence is perceived as not applying equally to all women [40]. Furthermore, women’s credibility is sometimes doubted [44]. Victim-blaming remains a widespread phenomenon: women are portrayed as having provoked, deserved, or even enjoyed the violence they were subjected to [45]. Equally, particularly in the case of rape and sexual assault, women are not believed [46]. A widespread rape-myth is that women make up accusations [47]. In this climate, women may be less willing to come forward and disclose incidents of violence, in fear of being blamed.

Attitudes towards gender-based violence against women are likely to hinder disclosure when these stigmatise and/or victimise victims. As Krug and colleagues [30] explain, in “countries with strong cultural pressures to keep violence behind “closed doors” or simply to accept it as “natural”, non-fatal violence is likely to be under-reported”. There are different thresholds at which, culturally, certain forms of violence may be perceived as justified and/or acceptable. Furthermore, attitudes on whether violence against women ought to be considered differently depending on the extent to which it is seen as a private matter or not may also hinder disclosure [1]. This is closely related to the idea of thresholds of violence, as incidents might be considered a private matter if they do not exceed culturally-defined boundaries beyond which public interventions become necessary [30]. Where violence is seen as a public concern, it might be easier for women to speak out about their experience. Conversely, non-intervention and the framing of violence as a private matter tends to increase incidence of violence against women [48].

Evidence on the role that alcohol plays in violence against women provides mixed findings. Some studies have pointed out limited evidence that alcohol use is a risk-factor [26]. Others have nonetheless pointed out the societal role played by alcohol use, such as disinhibition or impaired judgement, increasing aggressivity or using it as an excuse for bad behaviour such as for example in sexual violence [49, 50]. Societies in which the use and consumption of alcohol is higher may thus have higher levels of violence against women [1]. However, it is important to distinguish whether excessive alcohol consumption is happening at the household level, and therefore likely to play a more direct role in any episode of violence, or instead at societal level where the effect is more likely to be on what is permissible behaviour [51], and thus aligning with acceptable societal (gender) norms [46].

The levels of violent crimes in a society may be associated with higher levels of violence against women [1, 30]. This is likely to relate to attitudes that see either personal or community exposure to violence in a normalised way [52]. This has to do with how societies might valorise violence, such as for example glorifying certain forms as good forms of masculinity, or whether violence is accepted as a normal and legitimate form of behaviour [46].

Finally, the extent to which women are participating in the labour market can also affect the prevalence of violence [53–55]. Women transitioning and increasing their participation on the labour market can represent a challenge of gender norms and patriarchal family organisation [30]. Women’s employment can also challenge gender roles and notions of masculine dominance and honour [39] by appearing to undermine men’s economic power and decision-making [56]. Behavioural differences in different societies can lead to exposures to different risks, for example through employment outside the home, because of the opportunities it can afford in terms of lifestyles, leisure and socialisation [1]. Women involved in the labour market are also more at risk of sexual harassment, and this is particularly the case in industries and occupations that are dominated by men [37], including as a measure of control and domination when they access positions of authority [57].

### Situational factors

Participation in the labour market provides economic empowerment, and as such can challenge gender relations not only at societal level, but also in women’s direct environment [46]. This in turn can provide a backlash through the means of increased use of gender-based violence against women. The level of economic independence of women can also affect levels of violence experienced, such as for example when the presence of young children affects women’s ability to be economically independent and where they receive relatively lower incomes [46, 58, 59]. Low income, unemployment and low socio-economic status are generally associated with increased levels of violence [26, 30]. This is not to say that being relatively well-off protects from violence. In fact, violence cuts across all socio-economic classes [30, 60].

Exposure to violence against women in society is another factor linked to higher rates of disclosure [26, 61]. Low exposure to violence lowers its visibility, which can constrain voices as the responsibility is individualised and the incidents themselves not understood as a more structural phenomenon. For example, as a result of the #MeToo movement, the problem of sexual harassment/violence was exposed and made more visible, which in turn led to women both being able to put words on their experiences and gave them a platform/mechanism through which to disclose these incidents [62].

### Personal factors

A number of personal, largely demographic, factors are also related to the prevalence of violence against women. Attitudes towards violence can be shaped by different cultural and ethnic contexts, although it is important to address potential intersections without reinforcing racist stereotypes [46]. Belonging to a minority group can be related to lower income, but also fewer support mechanisms for women if they are living outside their country of origin. Age might also be a factor, in that younger women are likely to have greater awareness of violence, and greater propensity to see it as problematic. Younger women also socialise with other younger people that also hold more informed attitudes [46]. Other factors may play a role in increasing the prevalence of violence against women such as having a disability or homosexuality.

The most common perpetrator of sexual violence is often an intimate partner, leading to the conclusion that being married or cohabiting is a significant predictor of violence [30]. Findings also suggest that the ability to form new relationships is positively related to violence against women [8]. Women often understand or speak out about the violence only as they exit a relationship [45], after a period of time where the boundaries between courtship and coercion have become blurred [63]. The number of children in the household, particularly younger children, can also increase economic dependence and thus violence against women.

### Methodological factors

The methodological design of prevalence surveys can matter for the data they obtain and the estimates they produce. Prevalence surveys are difficult to conduct because of the sensitivity of the subject of violence, and typically, interviewers will undergo specialised training to equip them to deal with this topic. This calls for great care in how they are designed and implemented. Methodological critiques of the FRA survey raised questions on what are seen as inadequate sample size, especially for analysis of sub-sets; variation in methods of approaching respondents, by telephone in the Nordic countries, but not elsewhere; and the high variation in the response rate, specifically from 18.5% in Luxembourg to 84% in Hungary [64].

A first factor consider is contact method, as interviewees can be approached using different techniques. Whether first contact was made in person or over the phone can lead to different outcomes. For example, when first contact is made on the phone, it might be easier for the person to decline taking part compared to a face-to-face initial contact [4]. It might also be that previous contact by phone might pre-select women affected by violence and thus results in higher disclosure [64]. Given that the three countries in which the contact method was by phone are Denmark, Finland and Sweden, it is therefore important to include this factor when assessing the ‘Nordic Paradox’.

Another factor to consider are response rates. Women might not be equally interested in participating in a prevalence survey, depending on whether they have themselves been affected by the issue. Arguably, this can go either way. Women who have been victims of violence might be too affected or traumatised to talk about their experience. However, they might also want to avail of the opportunity to talk about their experience of violence. Empirical evidence suggests that higher response rates are associated with lower levels of disclosed violence [64]. In effect, higher response rates might dilute the number of disclosed incidents when more women take part.

## Materials and methods

### Data and measures

Despite increasing commitment at EU level to combat and prevent violence against women, the dearth of data that are harmonised and comparable at EU level has been a serious impediment to better understandings. A breakthrough in the development of measures of violence against women has been the EU-Wide Survey on Violence against Women carried out by the EU Agency for Fundamental Rights (FRA) in 2012 and released from 2014. The data are available from the UK Data Service, under Special Licence Access under the identifier 10.5255/UKDA-SN-7730-1. The study has been granted a Special License for secondary analysis (project number 125303) to the first author of this paper.

The survey was carried out in response to a request from the European Parliament for data on violence against women [65] and which was reiterated in the Council of the EU’s Conclusions on the eradication of violence against women in the EU [66]. It was launched in the Council of the EU on 5^th^ March 2014. One of the main aims of data collection is to provide a comparable, reliable and valid measure of the various forms of violence against women throughout the European Union (EU). The survey is the largest multi-country study on the topic and covered 42,023 women aged 18 to 74, with over 1,500 in all Member States but Luxembourg where the sample reached only just above 900 women. FRA’s survey represents the best effort to date to provide a comparable measure of violence against women in the EU. It delivers a comprehensive overview of prevalence rates with detailed breakdown and is therefore an invaluable tool for further analytical work. Several studies [7, 9, 67] have examined the validity and reliability of the measure of violence against women across the EU, including measurement invariance which ensures that the questions are interpreted in similar ways in different national contexts, although this is limited to violence perpetrated by intimate partners.

The FRA survey measures violence against women using two main sets of items that capture physical and sexual violence, adapted from the Revised Conflict Tactic Scale (CTS2) developed by Straus and colleagues [68]. Although the survey focuses on experiences of violence that took place both since the age of 15 and in the last 12 months, this analysis focuses only on the former or so-called ‘life-time’ prevalence measure. The analysis is based on experiences of violence that may arise from different perpetrators, including a current partner, previous partner or other persons, as opposed to solely intimate-partner violence. The breakdown of the variables used for the analysis, together with their original variable name and label is provided in Table 1.

**Table 1:** Variables used in EU-wide survey on Violence against women to measure prevalence.

Sometimes other people can do things that hurt you physically. In the next questions I would like to ask you about your experiences with persons other than your current or previous partner(s)/boyfriend(s)/girlfriend(s). Since you were 15 years old until now, how often has someone done any of the following to you.	Other persons	Current partner	Previous partner
Pushed you or shoved you?	D01b	E03b	G04b
Slapped you?	D01c	E03c	G04c
Threw a hard object at you?	D01d	E03d	G04d
Grabbed you or pulled your hair?	D01e	E03e	G04e
Beat you with a fist or a hard object, or kicked you?	D01f	E03f	G04f
Burned you?	D01g	E03g	G04g
Tried to suffocate you or strangle you?	D01h	E03h	G04h
Cut or stabbed you, or shot at you?	D01i	E03i	G04i
Beat your head against something?	D01j	E03j	G04j
Since you were 15 years old until now, how often has someone done any of the following to you:			
Forced you into sexual intercourse by holding you down or hurting you in some way? (By sexual intercourse we mean here forced oral sex, forced anal or vaginal penetration)	D05a	E04a	G05a
Apart from this, attempted to force you into sexual intercourse by holding you down or hurting you in some way? (By sexual intercourse we mean here forced oral sex, forced anal or vaginal penetration)	D05b	E04b	G05b
Apart from this, made you take part in any form of sexual activity when you did not want to or you were unable to refuse?	D05c	E04c	G05c
Or have you consented to sexual activity because you were afraid of what might happen if you refused?	D05d	E04d	G05d

Responses are coded using a categorical non-linear 4-point scale ranging from never (1), once (2), 2–5 times (3) to 6 or more times (4). Other possible values in the survey questionnaire include: Don’t know (DK = 7), Not Applicable (NA = 8) and Refused (RF = 9). In this analysis on overall lifetime prevalence of physical and/or sexual violence against women, this scale is simplified into a binary scale, coded as 0 when a woman has not experienced a given form of violence and 1 otherwise. Although this approach does not allow for a measure of the intensity of violence experienced by women in the EU, it provides an overview of the overall proportion of women that have experienced any form of violence. This approach has been commonly adopted across previous studies that use the same data source [6, 7, 9, 67].

Levels of gender equality are measured through EIGE’s Gender Equality Index for 2012. This specific year is used to control for time-related variance and to coincide with the timing of data collection of the FRA survey. The use of composite indicators measuring gender equality is widely used in the literature on violence against women [6, 34, 37, 69], including FRA itself [1]. Unlike the measurement of prevalence of violence against women, the validity of the measurement of gender equality using composite indicators appears to be largely accepted. Empirical studies of concurrent validity show a large degree of correlation between different measures, such as a correlation of 0.82 between EIGE’s Gender Equality Index and the World Economic Forum’s Global Gender Gap Index for EU Members States [5].

Data on prevalence and on levels of gender equality are complemented by data from different sources. The data linkage is made at the macro-level, i.e. countries. Eurobarometer 85.3 [70] provides information at the country level. These are public opinion surveys conducted by the European Commission twice a year since 1973, on a variety of topics. They cover a wide and representative range of the European population, including on attitudes towards violence against women. Careful planning goes into their design (e.g. questionnaire and wording of questions) as well as representativeness on the basis of age, sex or location. The quality of Eurobarometer is usually accepted as high, although its validity can be limited by respondents’ little knowledge or concern for the issue examined. Nonetheless, Eurobarometer data have been regularly used for scientific publications on a range of topics, including on gender and diversity [e.g. 71, 72, 73]. Eurostat is used as a source of information on crime, urban density or employment. Data from the European statistical system is strong when it comes to quality and harmonisation across countries. Information about drinking is derived from the WHO Global Health Observatory Data Repository, which also provides a source of high-quality harmonised data. The variables used in this analysis are summarised in Table 2.

**Table 2:** Variables used in the analysis.

Variable	Values	Year	Source
**Individual level**			
**No. of children under 18 in the household**	0; 1; 2; 3 or more	2012	FRA
**Cohabiting/married**	0: No; 1: Yes	2012	FRA
**In full-, part- or self-employment**	0: No; 1: Yes	2012	FRA
**Disability (self-reported)**	0: No; 1: Yes	2012	FRA
**Age**	1: 18–24; 2: 25–29; 3: 30–34; 4: 35–39; 5: 40–49; 6: 50–59; 7: 60+	2012	FRA
**Subjective economic situation**	1: Living comfortably on present income; 2: Coping on present income; 3: Finding it difficult on present income; 4: Finding it very difficult on present income	2012	FRA
**Sexual orientation**	0: Heterosexual; 1: Non-heterosexual	2012	FRA
**Education level**	1: Primary; 2: Secondary; 3: Tertiary	2012	FRA
**Citizen of the country of residence**	0: No; 1: Yes	2012	FRA
**In general, how common do you think violence against women by partners, acquaintances or strangers is in [your country]?**	1: Very common; 2: Fairly common; 3: Not very common; 4: Not at all common	2012	FRA
**Exposure to violence: Thinking about domestic violence against women—that is, violence involving partners or people who are in a relationship—do you know of any women who have been a victim of any form of domestic violence: In your circle of friends and family?**	0: No; 1: Yes	2012	FRA
**Exposure to violence: Thinking about domestic violence against women—that is, violence involving partners or people who are in a relationship—do you know of any women who have been a victim of any form of domestic violence: Where you work or study (or used to)?**	0: No; 1: Yes	2012	FRA
**Country level**			
**Contact method**	0: Visit; 1: Telephone	2012	FRA
**Response rate**	Numerical	2012	FRA
**Domestic violence should be handled as a private matter totally or tend to agree**	Numerical	2016	Eurobarometer 85.3
**Women often make up claims, totally or tend to agree**	Numerical	2016	Eurobarometer 85.3
**Violence against women is provoked by the victim, totally or tend to agree**	Numerical	2016	Eurobarometer 85.3
**Total alcohol per capita (15+ years) consumption (TAC) of pure alcohol**	Numerical	2008–2010	WHO Global Health Observatory Data Repository
**Violent crime rate in the population (violence against the person such as physical assault, robbery, and sexual offences including rape and sexual assault)**	Numerical	2012	Eurostat
**Full-time equivalent employment 15–64 women**	Numerical	2012	Eurostat
**Gender Equality Index 2012 (version 2017)**	Numerical	2012	European Institute for Gender Equality

### Analysis

Descriptive statistics were computed for all variables used from the EU-wide survey on violence against women using the weight ‘WtEUOverall’. At country level, this paper reproduces the figures provided from respective sources. To examine ‘disclosed prevalence’ in relation to other contextual variables, and taking into account the inbuilt variation across Member States, the analysis made use of multilevel modelling. Because the response variable is binary (that is it can only take one of two values) in nature, generalised linear multilevel models (GLMM) with a logit link function are used [74]. All independent variables were mean-centred for ease of interpretation:

$$\text{log}(\frac{\pi_{ij}}{1-\pi_{ij}})=\beta_{0}+\beta_{1}x_{1ij}+\cdots +\beta_{n}x_{nij}+\beta_{1}^{*}{\beta x}_{1j}^{*}+\cdots +\beta_{m}^{*}{\beta x}_{mj}^{*}+u_{j}+e_{ij}$$

with i women in j countries, where n is the number of individual level variables and m the number of country level variables, where *π*_*ij*_ = *P*(*y*_*ij*_ = 1) and with u the intercepts for each country and e the error term. During the model specification development, variance inflation factors (VIFs) were checked and were all well below the recommended threshold of 10 [75, 76]. The correlation matrix is presented in S1 File. This shows none or low correlation generally among individual-level variables. In contrast, methodological variables are positively associated with some country-level variables: there is lower response rate in countries with a higher violent crime rate and in countries with higher levels of gender equality, for example. Attitudes at the country-level are also highly correlated: believing that violence is provoked by the victim is positively associated with seeing violence as needing to be handled as a private matter or believing that women often make up claims.

## Results

### Descriptive statistics

According to the EU-wide survey on violence against women (Tables 3–5), the majority of women (63%) were either married or cohabiting. The majority (56%) of households did not have any children under the age of 18. Among those who did, 20% had one child under 18, 17% two children under 18, and 7% three or more children under 18. In relation to age, women were about evenly represented in each age category. Only a small proportion of women considered themselves to have a disability (4%), not to be heterosexual (2%), or not citizens of the country they lived in (3%).

**Table 3:** Individual level variables.

	No. of children under 18 in the household				Cohabiting or married	In full-, part- or self-employment	Disability (self-reported)
	0	1	2	3 +			
**Austria**	62%	21%	12%	5%	60%	54%	3%
**Belgium**	53%	20%	18%	8%	63%	53%	6%
**Bulgaria**	59%	22%	15%	4%	69%	49%	8%
**Croatia**	56%	20%	17%	7%	65%	37%	4%
**Cyprus**	63%	19%	12%	6%	63%	57%	1%
**Czech Rep**.	57%	19%	19%	6%	65%	50%	4%
**Denmark**	65%	13%	16%	6%	64%	53%	5%
**Estonia**	59%	23%	14%	4%	58%	53%	7%
**Finland**	66%	15%	13%	7%	67%	59%	3%
**France**	53%	19%	19%	9%	62%	56%	5%
**Germany**	61%	19%	15%	5%	59%	57%	5%
**Greece**	65%	14%	16%	4%	61%	42%	2%
**Hungary**	60%	19%	14%	7%	61%	42%	6%
**Ireland**	39%	22%	22%	17%	58%	41%	2%
**Italy**	64%	19%	12%	6%	62%	52%	2%
**Latvia**	57%	26%	13%	5%	59%	55%	4%
**Lithuania**	58%	24%	13%	4%	59%	49%	6%
**Luxembourg**	49%	21%	20%	10%	72%	58%	4%
**Malta**	59%	24%	14%	3%	68%	41%	3%
**Netherlands**	56%	15%	19%	10%	66%	59%	4%
**Poland**	49%	23%	19%	9%	66%	47%	3%
**Portugal**	56%	26%	14%	4%	61%	50%	5%
**Romania**	46%	26%	20%	9%	67%	38%	3%
**Slovakia**	55%	21%	18%	6%	63%	54%	5%
**Slovenia**	61%	21%	13%	5%	67%	49%	3%
**Spain**	56%	21%	17%	6%	65%	40%	5%
**Sweden**	62%	15%	18%	5%	62%	66%	5%
**UK**	49%	24%	19%	8%	63%	58%	6%

**Table 4:** Individual level variables.

	Age							Subjective economic situation			
	18–24	25–29	30–34	35–39	40–49	50–59	60+	Living comfortably on present income	Coping on present income	Finding it difficult on present income	Finding it very difficult on present income
**Austria**	11%	7%	10%	10%	22%	18%	21%	25%	59%	13%	3%
**Belgium**	12%	9%	9%	10%	21%	19%	21%	39%	43%	14%	4%
**Bulgaria**	12%	8%	11%	9%	18%	19%	24%	2%	29%	37%	32%
**Croatia**	11%	9%	10%	9%	19%	20%	23%	10%	32%	35%	23%
**Cyprus**	15%	14%	8%	10%	19%	16%	17%	17%	25%	32%	26%
**Czech Rep**.	12%	8%	12%	10%	17%	19%	22%	8%	44%	30%	19%
**Denmark**	12%	12%	5%	10%	20%	18%	23%	50%	39%	8%	3%
**Estonia**	13%	11%	8%	9%	18%	19%	23%	9%	51%	27%	14%
**Finland**	12%	8%	10%	8%	19%	20%	24%	33%	51%	13%	2%
**France**	12%	7%	10%	10%	20%	19%	21%	40%	39%	15%	7%
**Germany**	11%	7%	8%	8%	22%	19%	24%	17%	63%	16%	4%
**Greece**	10%	10%	9%	10%	20%	18%	23%	4%	21%	36%	40%
**Hungary**	11%	8%	11%	10%	17%	20%	23%	4%	34%	33%	29%
**Ireland**	13%	9%	16%	11%	19%	16%	16%	26%	42%	21%	10%
**Italy**	9%	8%	9%	11%	22%	18%	23%	32%	41%	16%	11%
**Latvia**	14%	9%	9%	9%	18%	18%	23%	5%	44%	33%	18%
**Lithuania**	14%	9%	9%	9%	20%	18%	22%	9%	45%	31%	15%
**Luxembourg**	11%	9%	11%	11%	22%	18%	18%	63%	26%	9%	2%
**Malta**	13%	8%	11%	9%	17%	19%	22%	23%	49%	21%	7%
**Netherlands**	12%	7%	10%	10%	22%	19%	21%	44%	42%	10%	5%
**Poland**	14%	10%	11%	9%	17%	21%	19%	8%	63%	23%	6%
**Portugal**	10%	10%	10%	10%	20%	18%	22%	13%	40%	32%	15%
**Romania**	14%	10%	10%	10%	18%	19%	20%	5%	39%	36%	20%
**Slovakia**	14%	10%	11%	10%	18%	19%	18%	12%	39%	32%	18%
**Slovenia**	11%	10%	9%	9%	20%	20%	21%	13%	44%	28%	15%
**Spain**	10%	11%	10%	11%	21%	17%	19%	30%	42%	18%	9%
**Sweden**	13%	7%	10%	10%	19%	18%	23%	42%	44%	11%	3%
**UK**	13%	9%	9%	10%	21%	17%	21%	38%	43%	14%	5%

Weighted data: ‘WtEUOverall’.

**Table 5:** Individual level variables.

	Not heterosexual	Education level			Not a citizen of the country of residence	How common is violence against women by partners, acquaintances or strangers				Knowing women victim of any form of domestic violence in circle of friends and family	Knowing women victim of any form of domestic violence: Where you work or study (or used to)
		Primary	Secondary	Tertiary		Very common	Fairly common	Not very common	Not at all common		
**Austria**	2.4%	21%	67%	12%	6%	22%	51%	24%	3%	28%	22%
**Belgium**	2.7%	22%	68%	10%	5%	27%	62%	10%	1%	45%	24%
**Bulgaria**	0.5%	27%	57%	16%	0%	26%	45%	21%	7%	27%	26%
**Croatia**	0.5%	34%	44%	22%	1%	42%	46%	9%	4%	39%	29%
**Cyprus**	1.2%	23%	51%	26%	1%	28%	42%	27%	3%	33%	18%
**Czech Rep**.	2.3%	11%	78%	10%	1%	12%	46%	36%	7%	18%	19%
**Denmark**	3.4%	8%	47%	45%	2%	11%	52%	36%	2%	43%	26%
**Estonia**	2.0%	16%	62%	22%	15%	12%	59%	28%	2%	41%	29%
**Finland**	3.3%	15%	58%	27%	0%	9%	56%	33%	2%	57%	30%
**France**	2.1%	40%	34%	27%	3%	31%	60%	9%	0%	53%	28%
**Germany**	0.3%	29%	59%	12%	3%	19%	55%	25%	1%	38%	20%
**Greece**	0.4%	34%	48%	18%	2%	20%	50%	28%	2%	37%	22%
**Hungary**	0.7%	20%	67%	14%	0%	18%	52%	25%	5%	26%	23%
**Ireland**	1.1%	26%	48%	26%	6%	36%	54%	9%	1%	42%	22%
**Italy**	0.7%	72%	11%	17%	2%	36%	56%	8%	1%	39%	19%
**Latvia**	0.9%	16%	56%	28%	11%	30%	53%	16%	1%	33%	22%
**Lithuania**	1.4%	13%	55%	32%	0%	35%	55%	10%	0%	52%	29%
**Luxembourg**	0.7%	27%	38%	34%	41%	20%	57%	22%	1%	47%	20%
**Malta**	2.6%	21%	62%	17%	1%	34%	58%	8%	0%	42%	23%
**Netherlands**	0.4%	25%	41%	34%	2%	26%	61%	10%	4%	44%	30%
**Poland**	0.7%	14%	64%	22%	0%	18%	50%	28%	4%	32%	18%
**Portugal**	0.2%	64%	25%	11%	2%	62%	34%	3%	0%	37%	19%
**Romania**	3.6%	50%	35%	16%	0%	33%	55%	11%	1%	30%	25%
**Slovakia**	0.4%	10%	75%	15%	2%	15%	54%	22%	9%	34%	31%
**Slovenia**	1.3%	29%	54%	16%	9%	30%	53%	16%	2%	39%	30%
**Spain**	4.8%	50%	31%	19%	2%	32%	55%	12%	1%	36%	17%
**Sweden**	2.3%	4%	52%	44%	6%	25%	58%	16%	1%	48%	30%
**UK**	2.4%	36%	36%	27%	6%	38%	49%	13%	0%	47%	25%

Weighted data: ‘WtEUOverall’.

Only 20% of women had been educated to tertiary level, with 37% and 43% respectively at primary and secondary level. Just over half of women (52%) were working (either employment or self-employment). The majority of women had good level of subjective economic well-being. A quarter (25%) felt they lived comfortably on their present income, with a further 46% feeling they coped. Nonetheless, a sizeable number of women found it difficult (19%) or even very difficult (9%) to cope on their present income. There was general recognition of how common violence against women by partners, acquaintances or strangers is in society: 28% of women felt that that it was very common and a further 54% that it was fairly common. Knowing victims of domestic violence against women was also high: 40% of women knew a victim in their circle of friends and family, and 22% a victim at work or at a place of study.

The results of the EU-wide survey on violence against women were complemented with other data to provide contextual information (Table 6). At country level, 16% of the population agreed or totally agreed that domestic violence should be handled as a private matter, 24% that women often make up claims, and 18% that violence against women is provoked by the victim. On average, alcohol consumption averaged 11 litres of pure alcohol per person aged 15 and above per year. The violent crime rate (reported instances per 100,000 inhabitants) ranged from 0.031 in Romania to 1.21 in the UK. Only 50% of women were in employment measured as full-time equivalent. Finally, the EU-28 average for the Gender Equality Index was 65 points out of 100.

**Table 6:** Country level variables.

	Contact method, face to face vs call	Response rate	Domestic violence should be handled as a private matter, totally or tend to agree	Women often make up claims, totally or tend to agree	Violence against women is provoked by the victim, totally or tend to agree	Total alcohol per capita (15+ years) consumption (TAC) of pure alcohol	Violent crime rate in the population (violence against the person such as physical assault, robbery, and sexual offences including rape and sexual assault)	Full-time equivalent employment 15–64 years old women	Gender Equality Index 2012 (version 2017)
**Austria**		57%	23%	27%	25%	10.3	0.57	53%	61.3
**Belgium**		34%	26%	23%	16%	11.0	1.08	47%	70.2
**Bulgaria**		59%	37%	23%	23%	11.4	0.10	56%	56.9
**Croatia**		48%	25%	24%	23%	12.2	0.20	45%	52.6
**Cyprus**		73%	30%	47%	31%	9.2	0.05	56%	50.6
**Czech Rep**.		47%	16%	28%	28%	13.0	0.18	56%	56.7
**Denmark**	Call	33%	6%	25%	16%	11.4	0.45	60%	75.6
**Estonia**		64%	15%	38%	43%	10.3	0.51	62%	53.5
**Finland**	Call	39%	16%	18%	11%	12.3	0.85	63%	74.4
**France**		27%	23%	15%	13%	12.2	0.42	54%	68.9
**Germany**		53%	10%	27%	21%	11.8	0.24	52%	64.9
**Greece**		70%	28%	25%	17%	10.3	0.08	40%	50.1
**Hungary**		84%	17%	25%	24%	13.3	0.38	51%	51.8
**Ireland**		48%	13%	26%	20%	11.9	0.22	46%	67.7
**Italy**		58%	11%	14%	11%	6.7	0.25	41%	56.5
**Latvia**		71%	33%	48%	64%	12.3	0.07	60%	56.2
**Lithuania**		48%	23%	48%	50%	15.4	0.09	60%	54.2
**Luxembourg**		19%	17%	31%	21%	11.9	0.85	51%	65.9
**Malta**		49%	23%	50%	43%	7.0	0.07	40%	57.8
**Netherlands**		27%	9%	17%	6%	9.9	0.74	45%	74.0
**Poland**		40%	23%	30%	31%	12.5	0.12	52%	56.9
**Portugal**		66%	15%	20%	12%	12.9	0.21	55%	54.4
**Romania**		55%	34%	25%	28%	14.4	0.03	51%	51.2
**Slovakia**		43%	26%	30%	37%	13.0	0.13	52%	52.4
**Slovenia**		44%	23%	28%	27%	11.6	0.12	58%	66.1
**Spain**		31%	15%	27%	10%	11.2	0.25	45%	67.4
**Sweden**	Call	20%	3%	9%	10%	9.2	1.19	64%	79.7
**UK**		37%	9%	34%	19%	11.6	1.21	51%	68.9

### Undoing the ‘Nordic Paradox’

The models used to examine the ‘Nordic Paradox’ in relation to the range of factors outlined above are presented in Table 7. Model 1 confirms previous findings that gender equality levels are positively related to the disclosed prevalence of violence against women (odds ratio = 1.04, p < 0.001), even when controlling for demographic factors. However, adding variables about methodological and other societal factors (Model 2) means that the effect is no longer statistically significant, although the difference in coefficients between the two models is only marginal. This suggests that differences in the prevalence of violence against women are influenced by other methodological and/or societal factors, besides levels of gender equality.

**Table 7:** Coefficients for prevalence of violence against women.

Predictors	Model 1		Model 2	
	Odds Ratios	p	Odds Ratios	p
**(Intercept)**	0.44	<0.001	0.42	<0.001
**No. of children under 18 in the household**	1.08	<0.001	1.04	0.002
**Cohabiting/married**	0.72	<0.001	0.74	<0.001
**In full-, part- or self-employment**	1.11	<0.001	1.03	0.280
**Disability (self-reported)**	1.02	0.038	1.01	0.128
**Age**	0.99	0.256	0.99	0.244
**Subjective economic situation**	1.39	<0.001	1.30	<0.001
**Sexual orientation**	3.26	<0.001	2.93	<0.001
**Education level**	1.06	<0.001	1.02	0.233
**Citizen of the country of residence**	0.75	<0.001	0.54	<0.001
**In general, how common do you think violence against women by partners, acquaintances or strangers is in [your country]?**			0.70	<0.001
**Exposure to violence: Thinking about domestic violence against women—that is, violence involving partners or people who are in a relationship—do you know of any women who have been a victim of any form of domestic violence: In your circle of friends and family?**			2.47	<0.001
**Exposure to violence: Thinking about domestic violence against women—that is, violence involving partners or people who are in a relationship—do you know of any women who have been a victim of any form of domestic violence: Where you work or study (or used to)?**			1.40	<0.001
**Contact method**			1.53	0.172
**Response rate**			1.00	0.798
**Domestic violence should be handled as a private matter totally or tend to agree**			0.98	0.127
**Women often make up claims, totally or tend to agree**			0.98	0.357
**Violence against women is provoked by the victim, totally or tend to agree**			1.02	0.139
**Total alcohol per capita (15+ years) consumption (TAC) of pure alcohol**			1.09	0.112
**Violent crime rate in the population (violence against the person such as physical assault, robbery, and sexual offences including rape and sexual assault)**			1.39	0.188
**Full-time equivalent employment 15–64 women**			0.24	0.429
**Gender Equality Index 2012 (version 2017)**	1.04	<0.001	1.03	0.112
**σ**^**2**^	3.29		3.29	
**τ**_**00**_	0.10 _country_		0.07 _country_	
**ICC**	0.03		0.02	
**N**	28 _country_		28 _country_	
**Observations**	38571		32988	
**AIC**	40968.386		35570.375	

This analysis also provides information on the relationship between a range of individual/socio-cultural factors and the disclosed prevalence of violence against women. Women are more likely to disclose experiences of violence where there are a greater number of children under 18 in the household (odds ratio = 1.04, p = 0.002), if they feel less well-off economically (odds ratio = 1.30, p < 0.001) or if they identify as non-heterosexual (odds ratio = 2.93, p < 0.001). On the contrary, women are less likely to disclose violence if they are a citizen of the country in which they live (odds ratio = 0.54, p < 0.001), or if they are currently married or cohabiting (odds ratio = 0.74, p < 0.001). Further, believing that violence against women is not common in the country of residence is associated with lower disclosure of violence (odds ratio = 0.70, p < 0.001). Relatedly, where women know a victim among their friends/family and at their place of work/study, this is associated with greater disclosure (respectively: odds ratio = 2.47, p < 0.001; odds ratio = 1.40, p < 0.001). Finally, it is noteworthy that none of the other socio-cultural factors considered—i.e. attitudes towards violence against women, alcohol consumption, employment rates or crime rates—are associated with disclosed prevalence.

### Towards an alternative ranking

To better understand the ‘Nordic Paradox’, we go back to the rankings established by the Fundamental Rights Agency where the Nordic countries such as Sweden and Finland appear to have the highest rates of violence against women. Any consideration of rankings need to bear in mind that these somewhat overstate differences between countries, when in fact studies based on latent means analysis show there is a large degree of overlap between the prevalence of violence against women across most EU countries [9, 67] and that country level of gender equality has little explanatory power in these rankings [69]. The results presented in Table 7 confirm that country-level variables are largely unrelated to the prevalence of violence against women. However, they clearly demonstrate the need to consider the effects of individual-level factors, as nested within different national contexts to shed further light on the ‘Nordic Paradox’.

The results of the multilevel models provide both an estimate of the coefficients for each factor but also a distinct intercept for each country. As each variable included in the model was mean-centred, it is possible to interpret these intercepts as the level of disclosed violence against women in each country, taking into account the variation associated with these same factors. The intercepts from the model can be interpreted as a ‘corrected’ measure of violence against women, when controlling for other factors, across the EU Members States. They range from approximately -0.5 to 0.5, with 0 standing for the average level of violence against women in the EU (Table 8). For example, Finland (0.03) has a ‘corrected’ prevalence level close to that EU-average. Ireland (-0.46) has the lowest level amidst all Member States, while the Czech Republic has the highest (0.45).

**Table 8:** Disclosed prevalence rankings comparison.

	Prevalence FRA	Rank FRA	Intercepts from Model 2	Rank Model 2
**Austria**	20%	27	-0.33	24
**Belgium**	36%	9	-0.01	17
**Bulgaria**	28%	16	0.09	14
**Croatia**	21%	26	-0.36	25
**Cyprus**	22%	22	0.20	7
**Czech Republic**	32%	13	0.45	1
**Denmark**	52%	1	0.42	2
**Estonia**	33%	12	0.11	13
**Finland**	47%	2	0.03	16
**France**	44%	5	0.36	4
**Germany**	35%	10	0.17	9
**Greece**	25%	20	0.15	11
**Hungary**	28%	16	-0.04	19
**Ireland**	26%	19	-0.46	28
**Italy**	27%	18	0.20	6
**Latvia**	39%	7	0.14	12
**Lithuania**	31%	14	-0.13	20
**Luxembourg**	38%	8	0.04	15
**Malta**	22%	22	-0.03	18
**Netherlands**	45%	4	0.37	3
**Poland**	19%	28	-0.44	27
**Portugal**	24%	21	-0.28	21
**Romania**	30%	15	0.21	5
**Slovakia**	34%	11	0.19	8
**Slovenia**	22%	22	-0.30	22
**Spain**	22%	22	-0.32	23
**Sweden**	46%	3	-0.36	26
**United Kingdom**	44%	5	0.16	10

It thus becomes interesting to see whether these ‘corrected’ levels of violence might be different from the original rankings provided by the Fundamental Rights Agency. These new rankings should not be understood as definite, as they can change together with the factors that are considered. Instead, they show that prevalence surveys—even high-quality ones such as the EU-wide Survey on Violence against Women—only provide a partial picture. As Table 8 reveals, taking into account a range of methodological, personal, situational and societal factors can provide radically different rankings.

**Fig 3:**
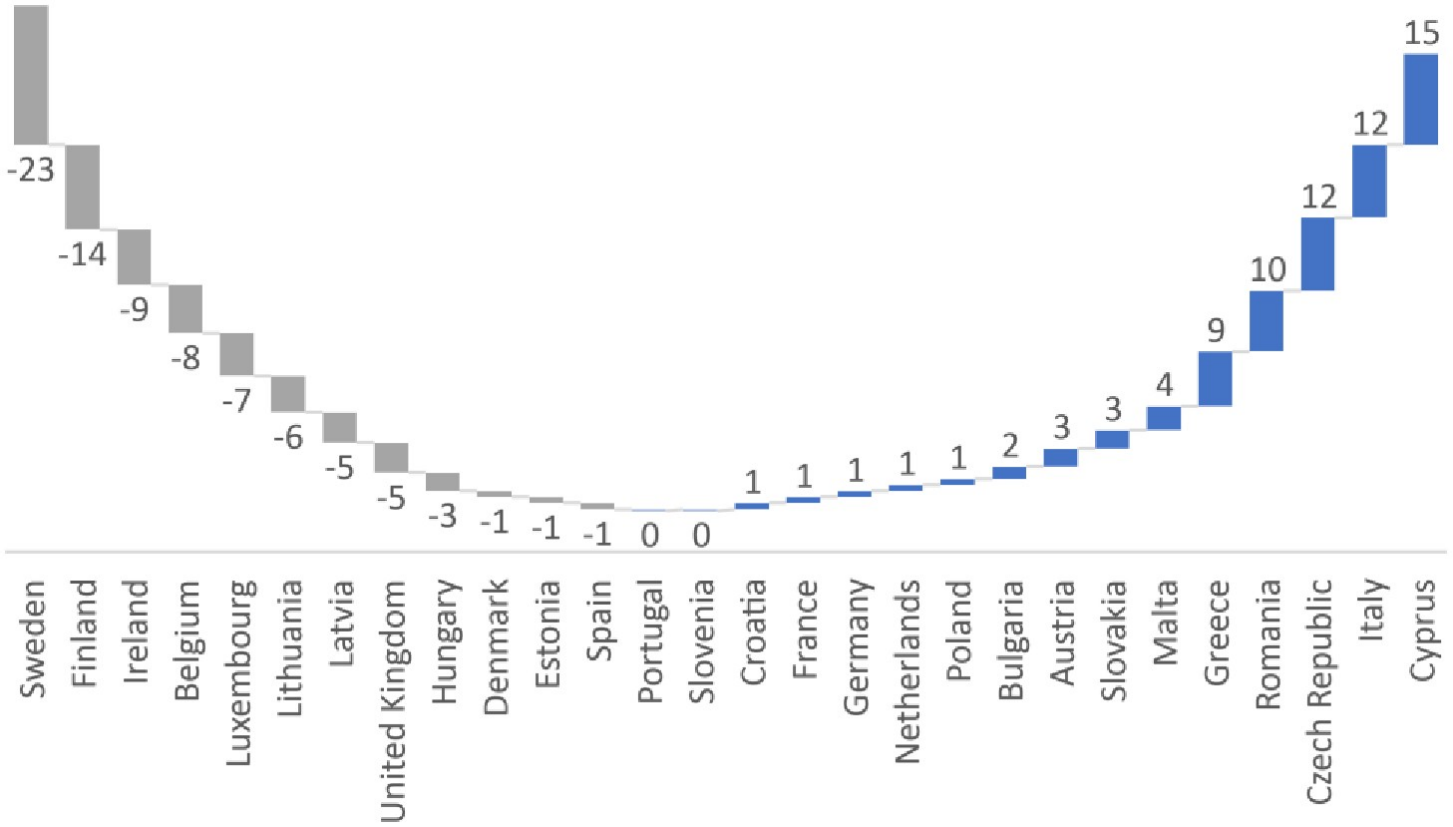
Differences in rankings between the FRA classification and those obtained from the models.

Fig 3 provides a visual representation of the changes between the two rankings. For nearly half the countries in the EU, there are little differences between the two (e.g. Croatia, Portugal, Austria, Denmark, Estonia, Hungary, Spain, France, Germany, Netherlands, Poland, Bulgaria or Slovakia) with at most a difference of three ranks. For others, controlling for other factors provides greatly different results. Using this method, there are higher levels of disclosed violence against women in EU countries such as Greece, Italy, Romania, the Czech Republic and Cyprus. Conversely, there is a dramatic drop in the rankings of Finland, and most particularly Sweden. This provides further evidence that the ‘Nordic Paradox’ can be ‘undone’ and better explained by relating it to societal and cultural factors.

**Fig 4:**
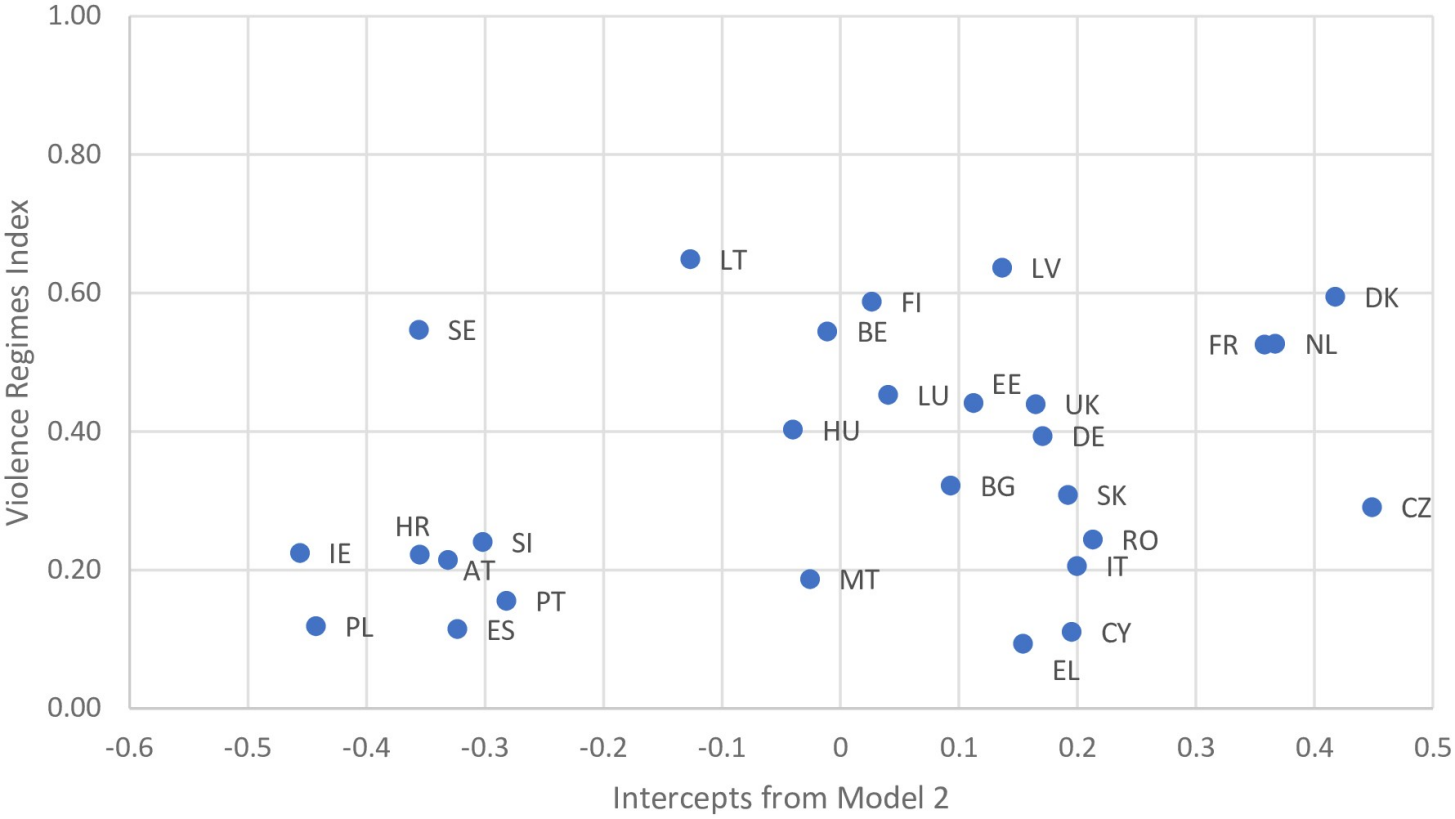
Relationship between adjusted levels of violence against women and the Violence Regimes Index.

**Fig 5:**
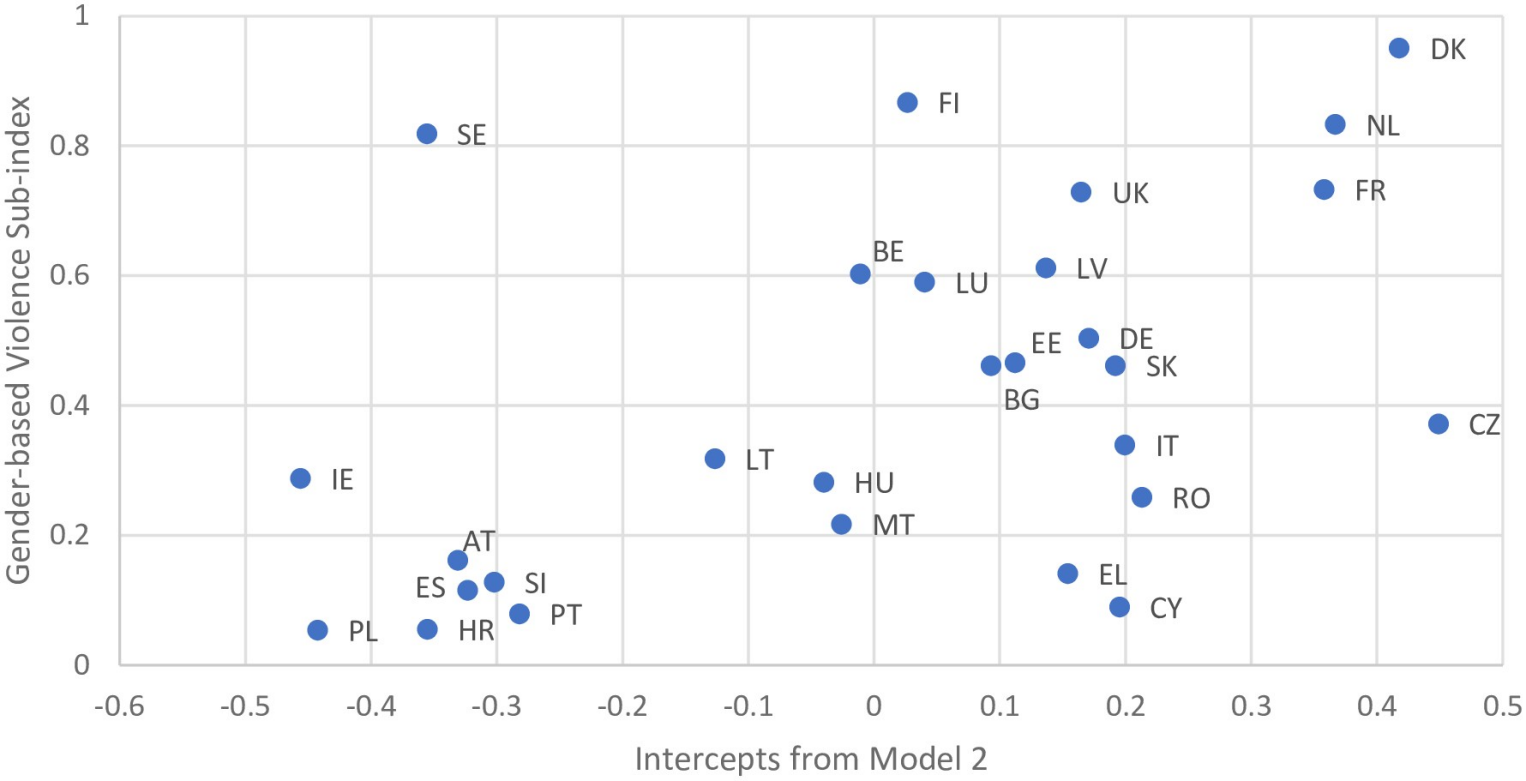
Relationship between adjusted levels of violence against women and the gender-based Violence sub-index (Violence Regimes Index).

Using these alternative rankings in relation to the Violence Regimes Index [25] shows moderate correlation between the two (r = 0.34), as illustrated in Fig 4. However, there is a stronger correlation (r = 0.51) with the Gender-based Violence sub-index (the Violence Regimes Index aggregates two sub-indices: deadly violence and gender-based violence). This is to be expected given that the two measures capture a similar concept, and use the same data source. However, the difference between the two is whether or not other factors are used. Fig 5 shows clearly that Sweden, Finland, the Netherlands and Denmark all score highly on the Gender-based Violence Sub-index. However, it also clearly shows that these countries fare very differently when other factors are accounted for, notably in the case of Sweden.

## Discussion

This paper has responded to calls to use multi-level modelling to better take into account the factors affecting disclosed levels of violence against women [6, 7], with the aim to also explore and try to understand the so-called ‘Nordic Paradox’. The results have shown that controlling for a range of other factors at the individual and country levels can provide an alternative ranking to that provided by the EU-wide Survey on Violence against Women, what we call undoing the ‘Nordic Paradox’. The results confirm the existence of a ‘Nordic Paradox’ but only when these other factors are not considered. We therefore conclude that the ‘Nordic Paradox’ is related to other factors that need to be examined to provide a more in-depth and nuanced understanding of the prevalence of violence against women in the EU.

In this paper, two types of factors proved to be of importance in explaining disclosed prevalence: exposure to violence in the environment particularly when women had first-hand exposure either in their direct family/friend environment, or in the wider circle of their place of work or study. This suggests that violence against women does not operate in isolation but instead in an ecosystem in which violence takes place. Being exposed to violence might also increase disclosure, as seeing it in the environment might normalise any experience of such violence, as something that happens also to others and may thus be verbalised.

It is interesting to note that perceptions of how common violence is in the country is negatively linked to disclosed violence. This is running contrary to exposure among family, friends or a place of work or study. This might be linked to the subjectivity of the question, as opposed to the more objective recall involved in asking about exposure in the environment, and speak to norms and expectations rather than actual incidents. Violence that is normalised and expected, when not linked to actual incidents and persons, can be dismissed as violence in itself. For example, where a slap by a partner is seen as common in society, this same slap might not be understood as an act of gender-based violence. This is known as the ‘process of normalisation’ [77].

The results showed that a number of personal factors such as dependent children, citizenship, sexual orientation or disability were related to disclosed prevalence. The strongest effect was for non-heterosexual women, who were about three times as likely to disclose violence than heterosexual women. This much higher prevalence, both by partners and non-partners, points to the possible use of violence as a form of hate crime, such as in extreme forms the use of ‘corrective rapes’ for homosexual women. There was also evidence that the position of the household mattered, for example economic dependence through the presence of children, but also as a citizen of the country in which women lived. Finally, results also showed that when comparative surveys do not employ the same methods across all countries, it is desirable to consider the effects of those differences on the overall results.

## Conclusion

The results of this analysis suggest that violence against women is not independent from a wider pattern of violence in society, but connected and co-constituted in specific formations, domains or regimes of violence [3, 25, 78]. This demonstrates the need to further examine how to define and conceptualise violence against women as well as violence more broadly, as violence is still often very much framed and defined in terms of direct and physical violence in order to measure and compare [2, 16], this even when feminist research has long recognised the continuum of violence against women [17, 19]. This paper shows that problematising measurement in relation to contextual factors raises the very question of “what is violence?” in an even more fundamental way; it problematises, pushes and challenges any simple definition and limits of violence. Instead, violence and violence against women need to be understood in relation to a set of societal conditions and structures of inequality; multiple contextual factors are relevant to conceptualising, measuring and explaining prevalence in violence and violence against women.

The implications of the study are that gender-based violence against women needs to be understood as a multi-level phenomenon, analysable in relation to socio-cultural conditions, as well as meso-situational, and (inter)personal level, rather than, say, only psychological or economic levels. The study also points to the importance of bringing together theoretical debates on violence and the difficult question of what violence is and what is included within the frame of violence, along with both multi-level analysis and statistical analysis. The paper shows that greater reliance on statistical modelling—and more appropriately the use of multi-level models—can provide new insights into the factors that may be related to disclosed prevalence of violence against women. Further work could go further by not only considering country level averages, but also incorporating heterogeneity around those means [69, 79].

Greater understanding on the effects of factors on prevalence, in turn, has got theoretical and policy implications. The factors related to disclosure can provide better understandings of how violence against women functions as a system, and can only lead to better policy responses. The findings of the greater risk for violence for women with dependent children, without local national citizenship, with non-normative sexual orientations, and with disabilities all highlight both major analytical and urgent policy and practice issues from an intersectional perspective. When the risk for violence increases with marginalisation, disadvantages or lack of privileges along multiple axes, policy and practice need to incorporate an intersectional understanding of gender applied in a policy context.

There are inevitably limitations in such a study as that reported here. Data are combined from multiple sources such as the FRA, WHO, Eurobarometer, Eurostat and EIGE. Thus, one set of limitations concerns the variable form, reliability and limitations of different datasets, the differences between prevalence surveys and other kinds of social data, as well as differences in these measures across time (different years) and place (meaning in this context, country). As discussed, debates about the FRA ranking are ongoing, with latent means studies showing a large degree of overlap in prevalence across most EU countries, but demonstrated measurement invariance such as for example in the case of IPVAW.

Another source of limitation concerns the perception of violence, and what counts as violence. When violence against women is perceived as more common, there is a tendency towards lower disclosure. This problematises absolute measurements of violence in the sense that the gap between disclosure and actual violence is likely related to this perception in the first place. Moreover, the measures used in this analysis do not capture women’s personal experiences but perceptions about their community. The link that can thus be established between these two different levels, while of relevance, are nonetheless a notable limitation.

Finally, in some societal and situational contexts, the normalisation of violence as well as previous violence and threats of violence may mean that further physical violence is not (necessarily) enacted. While the focus on acts of physical force is clearly vitally important, this may neglect forms of violence that are even less easily defined and measured, such as psychological, emotional and digital violence and abuse, as well as financial, health and social coercive controls.

## Supporting information

S1 File Correlation matrix. (DOCX)
