# Supplementary material for: Undoing the ‘Nordic Paradox’: Factors affecting rates of disclosed violence against women across the EU
Source: PLoS One. 2021 May 5;16(5):e0249693. doi: 10.1371/journal.pone.0249693 (PMC8099076; doi:10.1371/journal.pone.0249693)
Supplement: S1 File — (DOCX) [file pone.0249693.s001.docx]

## S1 File. Correlation matrix.

|  |  | 1 | 2 | 3 | 4 | 5 | 6 | 7 | 8 | 9 | 10 | 11 | 12 |
| --- | --- | --- | --- | --- | --- | --- | --- | --- | --- | --- | --- | --- | --- |
| 1 | Prevalence |  |  |  |  |  |  |  |  |  |  |  |  |
| 2 | Physical prevalence | 0.95 |  |  |  |  |  |  |  |  |  |  |  |
| 3 | Sexual prevalence | 0.51 | 0.38 |  |  |  |  |  |  |  |  |  |  |
| 4 | No. of children under 18 in the household | 0.03 | 0.03 | 0.02 |  |  |  |  |  |  |  |  |  |
| 5 | Cohabiting/married | -0.10 | -0.10 | -0.08 | 0.20 |  |  |  |  |  |  |  |  |
| 6 | In full-, part- or self-employment | 0.04 | 0.03 | 0.02 | 0.12 | 0.11 |  |  |  |  |  |  |  |
| 7 | Disability (self-reported) | 0.01 | 0.01 | 0.01 | -0.01 | -0.01 | -0.03 |  |  |  |  |  |  |
| 8 | Age | -0.03 | -0.04 | 0 | -0.26 | 0.08 | -0.18 | 0.02 |  |  |  |  |  |
| 9 | Subjective economic situation | 0.06 | 0.06 | 0.05 | 0.01 | -0.16 | -0.20 | 0.03 | 0.03 |  |  |  |  |
| 10 | Sexual orientation | 0.06 | 0.05 | 0.06 | -0.02 | -0.04 | 0 | 0 | -0.04 | 0 |  |  |  |
| 11 | Education level | 0.03 | 0.02 | 0.02 | 0.06 | 0.02 | 0.28 | -0.01 | -0.18 | -0.25 | 0.03 |  |  |
| 12 | Citizen of the country of residence | -0.02 | -0.02 | -0.02 | -0.04 | -0.01 | -0.02 | 0 | 0.04 | 0.01 | 0 | -0.02 |  |
| 13 | In general, how common do you think violence against women by partners, acquaintances or strangers is in [your country]? | -0.14 | -0.14 | -0.12 | -0.03 | 0.04 | 0.01 | 0 | 0 | -0.06 | 0 | 0.11 | -0.03 |
| 14 | Exposure to violence: Thinking about domestic violence against women – that is, violence involving partners or people who are in a relationship – do you know of any women who have been a victim of any form of domestic violence: In your circle of friends and family? | 0.24 | 0.23 | 0.18 | 0.06 | -0.03 | 0.05 | 0 | -0.07 | 0.01 | 0.03 | 0.05 | -0.01 |
| 15 | Exposure to violence: Thinking about domestic violence against women – that is, violence involving partners or people who are in a relationship – do you know of any women who have been a victim of any form of domestic violence: Where you work or study (or used to)? | 0.15 | 0.15 | 0.11 | 0.01 | -0.01 | 0.06 | -0.01 | 0.01 | 0 | 0.01 | 0.11 | 0.01 |
| 16 | Response rate | -0.10 | -0.09 | -0.08 | -0.05 | -0.03 | -0.07 | 0.02 | -0.02 | 0.34 | -0.05 | -0.11 | 0.06 |
| 17 | Contact method | 0.12 | 0.11 | 0.08 | -0.04 | -0.01 | 0.05 | 0 | 0.03 | -0.20 | 0.03 | 0.18 | 0.05 |
| 18 | Domestic violence should be handled as a private matter totally or tend to agree | -0.09 | -0.08 | -0.06 | 0 | 0.02 | -0.06 | 0 | -0.05 | 0.29 | -0.03 | -0.05 | 0.01 |
| 19 | Women often make up claims, totally or tend to agree | -0.05 | -0.05 | -0.03 | -0.01 | 0 | -0.03 | 0.01 | -0.06 | 0.13 | -0.04 | 0.07 | -0.07 |
| 20 | Violence against women is provoked by the victim, totally or tend to agree | -0.05 | -0.04 | -0.03 | -0.02 | -0.01 | -0.03 | 0.01 | -0.04 | 0.21 | -0.03 | 0.08 | -0.05 |
| 21 | Total alcohol per capita (15+ years) consumption (TAC) of pure alcohol | 0.01 | 0.02 | -0.02 | 0.03 | -0.01 | -0.04 | 0.01 | -0.01 | 0.14 | -0.01 | 0.02 | 0.02 |
| 22 | Violent crime rate in the population (violence against the person such as physical assault, robbery, and sexual offences including rape and sexual assault) | 0.12 | 0.11 | 0.10 | 0.01 | -0.01 | 0.09 | 0 | 0.04 | -0.32 | 0.05 | 0.10 | -0.08 |
| 23 | Full-time equivalent employment 15-64 years old women | 0.09 | 0.08 | 0.06 | -0.05 | -0.03 | 0.06 | 0.01 | 0.02 | -0.03 | 0.02 | 0.17 | 0 |
| 24 | Gender Equality Index 2012 (version 2017) | 0.13 | 0.12 | 0.10 | 0.02 | -0.01 | 0.08 | -0.02 | 0.03 | -0.38 | 0.05 | 0.13 | -0.03 |

|  |  | 13 | 14 | 15 | 16 | 17 | 18 | 19 | 20 | 21 | 22 | 23 | 24 |
| --- | --- | --- | --- | --- | --- | --- | --- | --- | --- | --- | --- | --- | --- |
| 1 | Prevalence |  |  |  |  |  |  |  |  |  |  |  |  |
| 2 | Physical prevalence |  |  |  |  |  |  |  |  |  |  |  |  |
| 3 | Sexual prevalence |  |  |  |  |  |  |  |  |  |  |  |  |
| 4 | No. of children under 18 in the household |  |  |  |  |  |  |  |  |  |  |  |  |
| 5 | Cohabiting/married |  |  |  |  |  |  |  |  |  |  |  |  |
| 6 | In full-, part- or self-employment |  |  |  |  |  |  |  |  |  |  |  |  |
| 7 | Disability (self-reported) |  |  |  |  |  |  |  |  |  |  |  |  |
| 8 | Age |  |  |  |  |  |  |  |  |  |  |  |  |
| 9 | Subjective economic situation |  |  |  |  |  |  |  |  |  |  |  |  |
| 10 | Sexual orientation |  |  |  |  |  |  |  |  |  |  |  |  |
| 11 | Education level |  |  |  |  |  |  |  |  |  |  |  |  |
| 12 | Citizen of the country of residence |  |  |  |  |  |  |  |  |  |  |  |  |
| 13 | In general, how common do you think violence against women by partners, acquaintances or strangers is in [your country]? |  |  |  |  |  |  |  |  |  |  |  |  |
| 14 | Exposure to violence: Thinking about domestic violence against women – that is, violence involving partners or people who are in a relationship – do you know of any women who have been a victim of any form of domestic violence: In your circle of friends and family? | -0.21 |  |  |  |  |  |  |  |  |  |  |  |
| 15 | Exposure to violence: Thinking about domestic violence against women – that is, violence involving partners or people who are in a relationship – do you know of any women who have been a victim of any form of domestic violence: Where you work or study (or used to)? | -0.14 | 0.29 |  |  |  |  |  |  |  |  |  |  |
| 16 | Response rate | 0 | -0.09 | -0.03 |  |  |  |  |  |  |  |  |  |
| 17 | Contact method | 0.11 | 0.06 | 0.04 | -0.39 |  |  |  |  |  |  |  |  |
| 18 | Domestic violence should be handled as a private matter totally or tend to agree | 0 | -0.05 | 0 | 0.39 | -0.47 |  |  |  |  |  |  |  |
| 19 | Women often make up claims, totally or tend to agree | 0.01 | -0.03 | -0.02 | 0.35 | -0.36 | 0.40 |  |  |  |  |  |  |
| 20 | Violence against women is provoked by the victim, totally or tend to agree | 0.04 | -0.04 | 0 | 0.43 | -0.32 | 0.53 | 0.86 |  |  |  |  |  |
| 21 | Total alcohol per capita (15+ years) consumption (TAC) of pure alcohol | 0.04 | -0.02 | 0.02 | 0.08 | -0.09 | 0.28 | 0.11 | 0.21 |  |  |  |  |
| 22 | Violent crime rate in the population (violence against the person such as physical assault, robbery, and sexual offences including rape and sexual assault) | 0.01 | 0.09 | 0.04 | -0.56 | 0.46 | -0.52 | -0.38 | -0.46 | -0.18 |  |  |  |
| 23 | Full-time equivalent employment 15-64 years old women | 0.11 | 0.02 | 0.04 | -0.09 | 0.55 | -0.11 | 0.02 | 0.20 | 0.39 | 0.23 |  |  |
| 24 | Gender Equality Index 2012 (version 2017) | 0.01 | 0.10 | 0.03 | -0.78 | 0.63 | -0.60 | -0.44 | -0.55 | -0.20 | 0.72 | 0.27 |  |
